# Impairment and restrictions in possibly benign multiple sclerosis

**DOI:** 10.1002/brb3.1259

**Published:** 2019-03-18

**Authors:** Laura Melanie Schaefer, Jana Poettgen, Anja Fischer, Stefan Gold, Jan‐Patrick Stellmann, Christoph Heesen

**Affiliations:** ^1^ Institute of Neuroimmunology and Multiple Sclerosis University Medical Centre Hamburg‐Eppendorf Hamburg Germany; ^2^ Department of Neurology University Medical Centre Hamburg-Eppendorf Hamburg Germany; ^3^ University of Central Lancashire Preston UK; ^4^ Klinik für Psychiatrie und Psychotherapie Charité Universitätsmedizin Berlin Berlin Germany

**Keywords:** cognition, multiple sclerosis, prognosis, quality of life

## Abstract

**Objective:**

The aim was to describe a broad range of health dimensions in possibly benign multiple sclerosis (MS) hypothesizing that despite some limitations there is a high adaptation to the disease.

**Methods:**

All patients from an outpatient university clinic data registry with an Expanded Disability Status Scale (EDSS) ≤3.5 and disease duration ≥15 years were addressed in a cross‐sectional study. Physical impairment, neuropsychological functioning but also influence on activities and patient reported outcome measures including coping were studied.

**Results:**

One hundred and twenty‐five patients could be included (mean EDSS: 2.8; mean disease duration: 24 years). Cognitive impairment was minor (8%) but fatigue (73%) and depression (46%) were prevalent. Nevertheless, QOL and daily activities seemed to be less affected. Patients showed high social support, coping abilities, and sense of coherence, which was predictive for their perceived benignity of the disease. Based on the EDSS alone, we estimated the rate of benign MS after 15 years of MS as high as 23% decreasing to 16% if cognition was included in the definition. However, cognitive performance was not relevantly associated with other outcomes.

**Conclusion:**

Common benign MS definitions seem to simplify a complex disease picture where different impairments and personal resources lead to more or less impact on people’s lives.

## 1 INTRODUCTION

Multiple sclerosis (MS) is a chronic, unpredictable disease with a broad variability in quality, severity, and evolution dynamics of symptoms (Degenhardt, Ramagopalan, Scalfari, & Ebers, 2009). Lately, the term benign multiple sclerosis (BMS) has been used to define patients with a milder disease course characterized by low disability assessed by the Expanded Disability Status Scale (EDSS) related to disease duration (Lublin & Reingold, 1996; Ramsaransing & De Keyser, 2006).

Based on different cutoffs prevalence estimates differ substantially in the few studies from 6%–74%. The strongest approach here is to reclaim no MS associated disability at all at life end classifying 5% of patients as benign (Skoog, Runmarker, Winblad, Ekholm, & Andersen, 2012). Taking also hidden MS symptoms as neuropsychiatric deficits into account the concept of a benign variant has in addition been questioned (Amato et al., 2006; Correale, Peirano, & Romano, 2012). Other studies claimed the predictive value of this BMS definition (Costelloe, Thompson, Walsh, Tubridy, & Hutchinson, 2008; Leray et al., 2013; Sayao, Bueno, Devonshire, & Tremlett, 2011). In the 2014 revision of MS disease course definitions, a consensus group advised to use the term BMS cautiously as even after years of a seemingly benign course the disease may decompensate (Lublin et al., 2014). In recent years, few efforts have been made to collect and describe putatively benign MS cohorts. However, with higher sensitivity of diagnostic criteria and increasing number of licensed treatments the open question is if all patients need to be treated as a benign variant of the disease might not exist. Taking it differently: do neurologist have the right to deny a possibly benign course of disease? The main aim of this study was to describe a broad range of health dimensions in relation to a BMS concept based on EDSS and disease duration. Special attention was payed to neuropsychological impairment as well as to coping and daily functioning. We hypothesized that despite of some limitations patients classified as BMS show a high level of adaption to the disease.

## 2 MATERIALS AND METHODS

### 2.1 Study design

MS patients fulfilling McDonald criteria (2005) were included in this cross‐sectional study who had presented at least once at the MS day hospital at the university medical center Hamburg between January 1996 and June 2012 and were considered having BMS based on a disease duration ≥15 years and an EDSS score ≤3.5 at their last examination. Patients were recruited by letter and gave their informed consent to the study. Patients were invited for an assessment at the center and received questionnaires in advance. We aimed to minimize a dropout bias as follows: In case patients were not able to take part in the assessment a structured telephone interview was performed. Patients who did not respond were contacted with a second letter including a feedback format for gathering information about their nonresponsiveness and general clinical status (stable, improved, worsen). The standardized assessment (July 2012–January 2013) included neuropsychological and physical function as well as nine questionnaires.

### 2.2 Clinical tests

Neurological impairment was assessed using the EDSS (Kurtzke, 1983). If patients could not take part in the assessment EDSS was evaluated by phone (Lechner‐Scott et al., 2003). In patients only answering the feedback letter and stating stability since their last examination we used their last EDSS. Mobility and ambulation was tested with three tests. The 25‐Foot Walk (T25FW) (Stellmann, Vettorazzi, Poettgen, & Heesen, 2014) is one of the best evaluated objective tests assessing gait impairment in a wide range in MS (Kempen et al., 2011). In addition the 3‐meter Timed Tandem Walk (TTW), (Stellmann, Vettorazzi et al., 2014) and the 6‐min Walking Test (6MWT)(Goldman, Marrie, & Cohen, 2008) were assessed. These tests are more sensitive to detect disability especially in mild affected patients and addresses additionally balance and fatigability (Kieseier & Pozzilli, 2012; Stellmann, Vettorazzi et al., 2014). Furthermore, the nine hole peg test (9HPT), for upper limb function was included.(Stellmann, Vettorazzi et al., 2014).

### 2.3 Neuropsychological assessment

Sixteen neuropsychological tests of approximately one hour examined memory, working memory, attention, and executive functioning: “Verbal Learning and Memory Test” (VLMT, verbal episodic memory), “repeating numbers” (ZN, numeric verbal memory), “Test Battery of Attention” (TAP, attention), oral “Symbol Digit Modality Test” (SDMT, information processing), “Regensburg Verbal Fluency Test” (RWT, semantic and phonematic verbal fluency), and executive functions with the “Performing Assessment System” (LPS) with subtests for logical reasoning and spatial perception. Results were adjusted for gender, age, and education. Z‐scores were calculated and we computed for each patient also a mean z‐scores over all tests as a global estimate of cognitive function. We displayed our data in different groups, representing different cutoff scores (<−2SD, <−1SD, <−1.65SD) in a specific proportion of tests (10%, 20%, 30%, 50% of the tests). However, our main definition classified patients as cognitive impaired if they scored 1.65 *SD* below the average (Rao, Leo, Bernardin, & Unverzagt, 1991) of a normal population in at least 20% of the tests (for references for neuropsychological assessments and questionnaires see Table S1).

### 2.4 Questionnaires

Nine questionnaires with a total of 195 items were applied. These comprised fatigue (fatigue scale for motor and cognitive functions, FSMC), depression (quick inventory of depressive symptomatology, QIDS‐SR16), cognition (multiple sclerosis neuropsychological questionnaire, MSNQ), QOL (Hamburg quality of live instruments in multiple sclerosis, HAQUAMS) activities of daily living (Frenchay activity index, FAI), leisure time activities (Godin Leisure time questionnaire, GLTQ), and demographic data. We asked for coping strategies using the short form questionnaire of the Coping and Self‐Efficacy Scale (CSES) which rates the extent of “one's confidence in performing coping behaviors when faced with life challenges”. Furthermore, the Sense of Coherence Scale was applied (SOC 29).

The “Sense of Coherence Scale of Antonovsky” (SOC) is based on the model of salutogensis, which centers the question what leads to health despite of what leads to illness. The Sense of Coherence is a “global orientation that expresses the extent to which one has a pervasive, enduring though, dynamic feeling of confidence that (a) the stimuli deriving from one's internal and external environments in the course of living are structured, predictable, and explicable; (b) the resources are available to one to meet the demands posed by the stimuli, and (c) these demands are challenges, worthy of investment and engagement”. Antonovsky called these three components comprehensibility, manageability, and meaningfulness and represented them in his scale as subcategories (Antonovxy, 1993). Finally, we asked patients to rate their disease as rather benign, neutral, or rather malignant.

### 2.5 Ethics

The Ethics Committee of the Hamburg Chamber of Physicians, Germany approved this study (Registration Number: PV4405).

### 2.6 Statistical analysis

For statistical analysis we used SPSS 19 (spss.com) and R (r‐project.org). Depending on the nature of the data we report descriptive statistics as mean/sd, median/range, or frequencies. We applied *t* test and respectively a Person chi‐square test to compare study and dropout patients. To analyze the association between outcomes, we used linear models or Fisher's exact test. We extracted R‐squared from significant models to quantify the strength of associations. For plotting, missing R‐squared values from significant Fisher's exact tests were set arbitrarily to a fixed low value of 0.2. All *p*‐values were corrected for multiple testing with the false discovery rate and were considered significant if still below 0.05. We analyzed the impact of different outcomes on QOL, the ability to work and the patient rated severity of their disease in multivariate models that underwent a stepwise selection of variables based on the Akaike Information Criterion (Akaike, 2011).

## 3 RESULTS

### 3.1 Cohort

**Figure 1:**
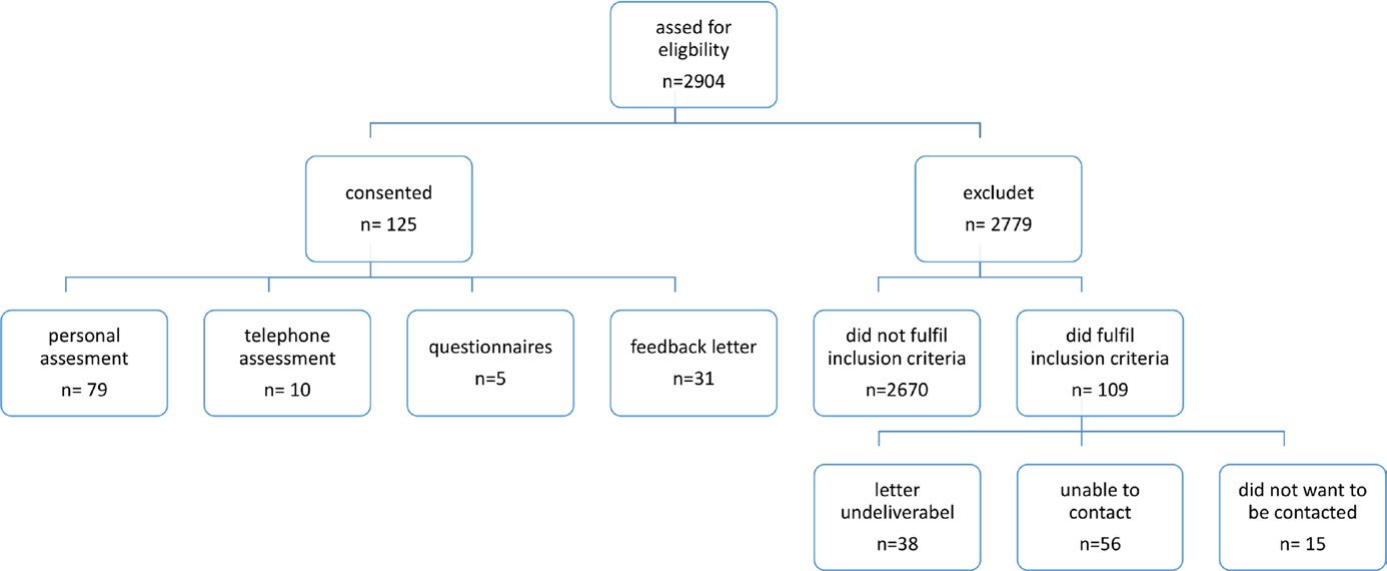
Composition of the cohort. *n* = 879 MS database with EDSS <4, Cohort *n* = 125, dropouts = 2,779

Out of 2,904 patients from the database, 879 (30%) had at least 15 years of disease duration while 234 patients (8.1%) fulfilled also the inclusion criteria for possibly BMS with an EDSS ≤3.5. Mean EDSS was 2.5 ± 0.9 with a disease duration of 23.4 ± 6.2 years (mean, *SD*). One hundred and twenty‐five patients (53% of 234) could be contacted and built the actually studied cohort*.* Seventy‐nine patients performed clinical assessment including neuropsychological examination, 10 patients were interviewed by phone and five patients just filled in questionnaires (for see study flow‐chart; Figure 1). Thirty‐one patients just replied with a short feedback letter leading to *n* = 125 with basic MS demographic data. There were no significant differences between the cohort and the dropouts based on the most recent EDSS score (*p* = 0.58), disease duration (*p* = 0.08), age (*p* = 0.07), and gender (*p* = 0.42). Only the time since the last EDSS examination was on average 1.2 years (*p* < 0.01) shorter in the available cohort.

### 3.2 Demography

Included patients were on average 51 years old and had a female: male ratio of 3:1. Most patients (65%) had relapsing‐remitting MS (RRMS) and had never (42%), or less than five years (37%) been treated with immunotherapy. Twenty‐one per cent had been treated for more than five years including 2% with escalation therapies. At the time of assessment 37% patients were on immunotherapies. Mean EDSS was 2.8 (*SD* 0.99) including 15% patients with an EDSS >3.5. From all EDSS Scores (*n* = 106, Median 2.5 (Range 0–6)), 79 patients were examined due to the study (Median 2.5; Range 0–6) and further nine patients in the MS‐day clinic during the study time (Median 2.5 (1–3.5). In addition, nine patients were scored by telephone‐EDSS (Median 3.0 (2–4). Nine patients stated to feel stable and we used their last EDSS (Median 2.4, Range 1–3.5). The most affected functional systems were the pyramidal (mean 1.3, *SD* 1.1), the sensory (mean 1.39, *SD* 0.94), and the cerebral (mean 1.14, *SD* 0.93) system (*n* = 92). Thirty‐nine per cent of patients had an unlimited walking distance. Assuming the same EDSS distribution in dropouts, the estimated rate of BMS defined by EDSS ≤3.5 based on all patients with disease durations>15 years (*n* = 879) from our database was 22% (199 out of 879; 95%CI: 19.8%–25.4%). The mean 6MWT distance was 466 meters and patients needed five seconds for the 25FWT and 12 s for the TTW.

The majority had more than 10 years of education (59%), lived in a partnership (75%), and had children (59%). Thirty‐three per cent were working fulltime, while 20% were retired due to illness. All results are summarized in Table 1, for further details see Table S2.

**Table 1:** Demography

	*n* (%)^a^	*n*
Sex (f:m)	93:32 (74%: 26%)	125
Age, mean (*SD*)	51.11 (8.87)	125
Disease duration^d^, mean years (*SD*)	24.04 (6.89)	125
Disease courses		
RRMS	60 (65%)	94
SPMS^b^	23 (25%)	
PPMS^c^	5 (5%)	
Unknown	5 (5%)	
Medication		
Never	39 (42%)	94
<5 years	32 (37%)	
>5 years	20 (21%)	
Walking distance		
Unlimited	36 (39%)	94
>1,000	29 (32%)	
500–1000 m	23 (25%)	
300m	4 (4%)	
Prestudy EDSS score, mean (*SD*)	2.5 (0.87)	125
Last EDSS examination		
Mean (*SD*) years ago	2.7 (1.75)	125
Actual EDSS^e^		
Total score, mean (*SD*)	2.8 (0.99)	106
Median (range)	2.5 (0−6)	
EDSS ≤2.0	36 (29%)	
EDSS 2.5−3.5	69 (56%)	
EDSS >3.5	19 (15%)	
Motor function		
9HPT right, mean seconds (*SD*)	20.21 (0.43)	79
9HPT left mean seconds (*SD*)	21.66 (0.57)	
25FWT, mean seconds (*SD*)	5.22 (1.52)	
TTW mean seconds (*SD*)	11.52 (5.68)	
6MWT mean meter (*SD*)	465.81 (122.91)	

6MWT: 6‐min Walking Test; 9HPT: nine‐hole peg test; 25FWT: 25‐Foot Walk; EDSS: expanded disability scale; TTW: Timed Tandem Walk.

a If not other indicated *n* (%).

b Secondary progressive MS.

c Primary progressive MS.

d Since first symptoms.

e *n* = 106 (median = 2.7; range 0–6) = study‐EDSS *n* = 79 (median 2.5; range 0–6) + *n* = 27 (median 3; range 1–4).

### 3.3 Neuropsychological assessment

In general, the z‐scores of memory, working memory, attention, and executive function tests were within the normal range. Most cognitive deficits were found in the domains of attention (5%–18%), short term (9%), and working memory (8%) as well as word fluency (6%–10%).

In summary, 8% of the patients scored 1.65 *SD* below average in more than 20% of the cognitive tests and were categorized as cognitively impaired. Using a cutoff score <−2 *SD* in more than 10% of the tests 14% of the patients were affected. Twenty‐eight per cent of the patients scored <−1 *SD* in more than 30% of the cognitive tests (Table 2). Sixteen patients with an EDSS below 4 were cognitively impaired resulting in 59.5% of BMS cases if BMS was defined by EDSS and cognition. Concerning the whole dataset, the corresponding rate of BMS corrected for cognitive impairment was 15.8% (139 out of 879, 95%; CI: 13.4%–18.2%).

**Table 2:** Neuropsychological outcome

Meaning	Test name	Mean (*SD*)	Affected *SD* <−1.65, %	Severely affected *SD* <−2, %	Moderately affected *SD* <−1 and ≥−2, %	Not affected *SD* ≥−1, %
Memory						
Memory span	VLMTS1	0.24 (1.04)	3	0	8	92
	ZNfw^a^	0.45 (1.23)	3	3	5	92
Learning	VLMTS1‐5	0.28 (0.92)	4	1	6	93
Short Term Memory	VLMT5‐7	−0.29 (0.95)	9	5	13	82
Recognition	VLMTW‐F	−0.10 (0.93)	5	4	11	85
Working memory	SDMT	0.14 (1.02)	8	1	10	89
	ZNbw^b^	0.04 (1.07)	4	4	20	76
Attention						
Alertness	Tonic	−0.83 (0.77)	11	3	34	63
	Phasic	−0.89 (0.79)	9	2	46	52
Selective attention	GoNoGo	−0.39 (0.93)	5	4	18	78
Divided attention	Visual	−0.25 (1.1)	11	8	15	77
	Acoustic	−0.7 (0.99)	18	11	24	65
Executive function						
Verbal word fluency	Semantic	0.69 (1.4)	6	4	4	92
	Phonematic	−0.09 (1.23)	10	6	24	70
Logical reasoning	LPS3	0.74 (0.50)	0	0	0	100
Spatial perception	LPS7	0.60 (0.70)	0	0	1	99
Score						
More than 50% tests abnormal			0	0	2.5	
More than 30% tests abnormal			5	1	28	
More than 20% tests abnormal			8	3	35	
More than 10% tests abnormal			25	14	60	

*n* = 79.

VLMT: “Verbal Learning and Memory Test”; ZN: repeating numbers Test; SDMT: “Symbol Digit Modality Test”; RWT: Regensburg Verbal Fluency Test; LPS: Performing Assessment System.

a Forward.

b Backward.

### 3.4 Questionnaires

Results from the questionnaires are summarized in Table 3. Based on MSNQ, 27% of the patients rated themselves as cognitively affected. Mean QIDS score of 6.31 (*SD* 4.53) indicated mild depressive symptomatology. One‐third of the patients had low grade depressive symptoms, 9% moderate, and 7% severe depression. Seventy‐three per cent showed pathological FSMC total scores including 43% patients with severe fatigue. The three reported main symptoms in the HAQUAMS were walking difficulties (33%), fatigue (20%), and sensory symptoms (15%). Asked for their overall QOL patients mean score on a single fife point Likert scale item was 3.51 which means “quite satisfied”. The CSES total score (possible range 0–10 with higher values indicating higher coping abilities) showed a mean of 6.38 (*SD* 2.19) with the social support subcategory scoring highest. The SOC mean score of 5.1 (*SD* 0.84) was similar to healthy population data (Schumacher, Wilz, Gunzelmann, & Brähler, 2000). The main activities which patients did not perform at all according to the FAI were “gardening” (40%), “travel outing/car ride” (21%), and “heavy household work” (13%) (Table S3). The GLTQ indicated that the cohort did in average 0.66 times per week light, 1.74 times per week moderate, and 1.75 times per week strenuous exercise. Seventy‐five (76%) patients estimated their MS form as benign, six (8%) as malignant, and 12 (16%) as neutral.

**Table 3:** Patient reported outcome measures (*n* = 94)

	Mean (*SD*)	Mean/question (*SD*)
MSNQ		
Total score	18.52 (9.25)	1.23 (0.62)
QIDS16		
Total score	6.31 (4.53)	0.70 (0.50)
FSMC		
Total score	57.43 (21.5)	2.87 (1.07)
Cognitive fatigue	27.34 (11.25)	2.73 (1.13)
Motor fatigue	30.1 (11.07)	3.01 (1.12)
CSES		
Total score	82.87 (28.58)	6.38 (2.19)
Problem focused	39.32 (13.96)	6.55 (2.33)
Emotion focused	22.21 (11.06)	5.55 (2.77)
With social support	21.34 (6.94)	7.12 (2.31)
SOC		
Total score	146.8 (24.45)	5.1 (0.84)
HAQUAMS		
Total score		2.06 (0.64)
Fatigue		2.29 (1.11)
Cognition		2.30 (1.08)
Lower extremity		2.21 (0.86)
Upper extremity		1.46 (0.60)
Communication		1.97 (0.88)
FAI		
Total score	31.19 (6.68)	2.08 (0.45)
GLTQ		
Score	19.55 (20.55)	

Note

CSES: Coping and Self‐Efficacy Scale; FAI: Frenchay activity index; FSMC: fatigue scale for motor and cognitive functions; GLTQ: Godin Leisure time questionnaire; HQUAMS: Hamburg quality of live instruments in multiple sclerosis; MSNQ: multiple sclerosis neuropsychological questionnaire; QIDS‐16: quick inventory of depressive symptomatology; SOC: Sense of Coherence Scale.

### 3.5 Associations

**Figure 2:**
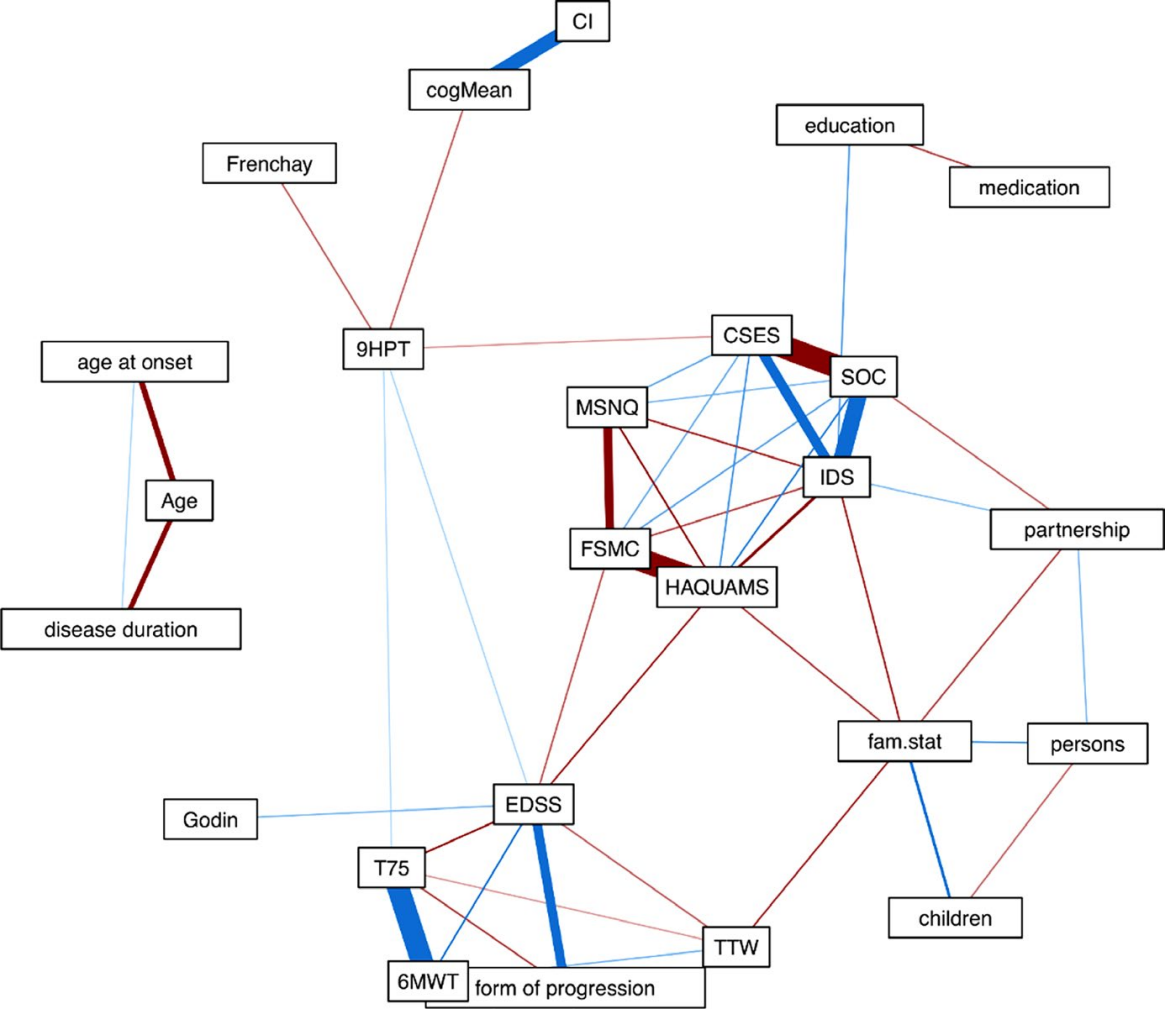
Correlations between outcomes. Red color stands for negative correlation, blue for positive correlations. The thickness of the lines pictures the strengths of the correlation. Just significant correlations after correction for multiple testing are shown. CI: cognitive impairment; cogMean: computed mean *z*‐score of all neuropsychological tests; 9HPT: Nine‐Hole Peg Test; CSES: Coping Self‐Efficacy Scale; SOC: Sense of Coherence; IDS: Quick Inventory of Depressive Symptomatology, QIDS‐SR16; MSNQ: Multiple Sclerosis Neuropsychological Questionnaire; HAQUAMS: Hamburg Quality of Live Scale in Multiple Sclerosis; FSMC: Fatigue Scale for Motor and Cognitive Functions; EDSS: Expanded Disability Status Scale; TTW: Timed Tandem Walk; T75: 25‐Foot Walk; Godin: Godin leisure time activities

The association and dependencies between outcomes are summarized in Figure 2. We observed approximately four clusters: EDSS/mobility (TTW, T25FW, 6MWT, 9HPT), neuropsychology, family status, and PROMS. However, there were only few links between the clusters (Figure S1). QOL assessed with the HAQUAMS had a prominent position within the network of associations bridging between disability measures as EDSS or fatigue and family status, coping, and mood. Interestingly, cognitive impairment (summarized as mean‐z score) and immunotherapies were rather independent from other outcomes. Furthermore, there was no difference concerning EDSS, SDMT, and Fatigue between patients with and without actual immunotherapies. Age and disease duration were not related to any other measurement.

### 3.6 Multivariate models

To elucidate, what determines QOL, we investigated the impact of disease duration, coping, EDSS, cognition, fatigue, medication, ability to work, and depression. After stepwise selection of variables, the HAQUAMS score was substantially explained (R^2^=0.68) by EDSS (*p* = 0.001), FSMC (*p* < 0.001), IDS (*p* < 0.001), and occupational situation (*p* = 0.038). In this context, we analyzed also the difference in QOL comparing differently defined BMS groups and observed a significant better QOL in BMS patients defined by EDSS alone (*p* = 0.014) while BMS groups defined by cognitive impairment and EDSS did not differ in QOL (*p* = 0.15). The ability to work was weakly explained by the HAQUAMS score alone (R^2^ = 0.11, *p* = 0.010), while disease duration, coping, EDSS, 6MWT, NHPT, cognition, depression, or fatigue did not contribute. Patients rating of severity of their MS depended (*p* = 0.007) on coping and cognition. Lower coping scores were associated with a rating of MS as a severe condition. QOL, disease duration, EDSS, 6MWT, 9HPT, Fatigue, medication, and depression did not contribute to the rating. Patients with a cognitive impairment avoided to rate their disease as benign or malignant and favored the neutral response. See Figure 2 (Figure S1).

## 4 DISCUSSION

Studying a cohort of presumably benign MS we found restrictions in motor function, fatigue, and depression but with only moderate influence on patients QOL. Cognitive deficits showed a striking low prevalence. Seventy‐five per cent of the patients rated their MS themselves as benign. Depending on the inclusion of cognitive impairment in the BMS definition, the rate of BMS in our dataset of long standing MS (*n* = 879) ranged between 16% and 23%.

Interestingly, although having applied a MS sensitive neuropsychological battery only 8% of patients had a substantial cognitive deficit. The few studies addressing cognitive impairment in BMS show a large variance from 17% to 47% (Amato et al., 2006; Correale, Ysrraelit, & Fiol, 2012; Gajofatto, Turatti, Bianchi, & Forlivesi, 2015). Cognitive impairment that is common in MS, seems to be independent from other disability dimensions and might indicate a higher risk for later disability progression (Correale, Ysrraelit et al., 2012; Portaccio et al., 2009; Rao et al., 1991; Sayao, Devonshire, & Tremlett, 2007). However, different neuropsychological batteries and cutoff scores to define cognitive impairment restricts comparability of studies (Fischer et al., 2014). While most studies define two‐three tests scores below −2 *SD* of a normal population as cognitive impairment, the ecologic validity of such a definition for impairment in daily life remains a matter of discussion (Gajofatto et al., 2015). Here, we observed also no relevant association between cognitive performance and QOL or FAI.

In contrast, 73% of our patients indicated a substantial amount of fatigue that is above other BMS studies reporting 33% to 54% of affected patients (Amato et al., 2006; Correale, Peirano et al., 2012; Sayao et al., 2011). Fatigue scores were closely associated with depression and coping and thus contributing to QOL. However, HAQUAMS mean scores were still 0.15‐0.42 points lower than in other MS cohorts meaning a better QOL. Given a minimal important difference of 0.2 points, our results indicate a preserved high QOL in our cohort (Gold et al., 2010; Schäffler et al., 2013). Thus, even a high prevalence of high FSMC fatigue scores did not severely impact on the QOL of our patients. Similar, daily activities as assessed by FAI showed high functionality above for example a population based MS cohort in Stockholm (Einarsson, Gottberg, Fredrikson, von Koch, & Holmqvist, 2006). Still 20% of our cohort stated to be retired due to MS. Sayao et al. also found a higher QOL and higher employment rates in long‐term BMS patients than in those not staying benign (Sayao et al., 2011). In our cohort, we identified QOL as an exclusive but very weak predictor for employment status.

Overall, QOL showed strong associations with a broad range of health dimensions. As contributing factor for a high QOL, we found a supporting background, effective coping strategies as well as a good sense of coherence. Especially, the patients’ impression of a benign disease was associated with better coping abilities. Most patients reported a high level of coping self‐efficacy. Social support was the strongest contributive factor. In addition, most of our patients lived in a partnership and had children which is in contrast to previous observations in the general MS population reporting higher divorce rates (Pfleger, Flachs, & Koch‐henriksen, 2010) and lower pregnancy rates.(Alwan, Chambers, Armenti, & Sadovnick, 2015) Our findings indicate a high level of social integration and support in our sample. In addition, Sense of Coherence (SOC) scores were overall high and (Eriksson & Lindström, 2005) only 8% of our patients scored 1.65 *SD* below the average scores from a population‐based study in healthy German people (*n* = 2005) (Schumacher et al., 2000). Thus, our MS patients resembled healthy individuals in their perception of meaningfulness of life.

At the time of the actual examination about 15% of the patients showed EDSS scores ≥3.5 and only 39% reported an unlimited walking range. Correspondingly, other motor‐focused objective assessments as the TTW (Stellmann, Vettorazzi et al., 2014) and the 6MWT (Goldman et al., 2008) showed impairment. Among all objective outcomes, mobility restrictions contributed highest to the QOL underlining previous reports about the importance of walking abilities for MS patients (Heesen et al., 2017). In contrast, having had immunotherapies did not seem to influence any of our outcomes including QOL.

In our cohort, 30% of patients had some sort of progressive disease course. While a consensus group defined BMS independently of the disease course phenotype (Lublin et al., 2014), Skoog et al. proposed the absence of progression as a condition to define BMS (Skoog, Tedeholm, Runmarker, Odén, & Andersen, 2014). However, even primary‐progressive MS patients presumed to have a worse prognosis show a heterogeneous disease evolution. Therefore, we decided against a paradigmatic exclusion of a possibly benign progressive disease course (Stellmann, Neuhaus, Lederer, Daumer, & Heesen, 2014). Here, we observed only a moderate association between the disease course and disability while QOL or FAI were independent from the disease course.

As a limitation nearly half of the patients could not be contacted and only a third could be assessed clinically. But baseline demographic data of these compared to the analyzed cohort gave no indication of a selection bias. In addition, we hypothesized that especially minor impaired MS patients might not seek medical attention at a tertiary referral clinic. Thus, a negative selection bias might rather lead to an overestimation of impairment in the clinically investigated cohort. Even though the EDSS of the majority of patients were clinically assessed, we used also in some cases retrospective data which is a further limitation. Furthermore, this study included no healthy control cohort but referred to normative data from the literature which weakens the validity of findings to some extent.

In conclusion, existence and prevalence of BMS is a heavily disputed scientific topic (Amato & Portaccio, 2012; Lublin, 2014) and our data add to the complexity of the picture. Presumably benign patients seem to have some impairment as especially walking restriction and fatigue but most patients live their lives as they want to. Seventy‐five per cent of the patients rated their MS as benign reflected in high SOC and QOL scores.

Thus we propose that BMS needs to be defined at least partially by educated patients themselves based on their estimates how far MS impacts their life goals and impairs their ability to adapt to life challenges. This view might help to stress rather resources and resilience than clinical deficits. Highly sensitive disability measures as for example neuropsychological batteries have a questionable value for a given patient and might not be the best approach to define “benign” in a patient centered way.

## CONFLICT OF INTEREST

C. Heesen has received research grants, speaker honoraries, and travel grants from Biogen, Genzyme, Merck, Teva, Roche, Sanofi‐Aventis, Bayer. J. P. Stellmann was a National MS Society (US) Postdoctoral Fellow for Rehabilitation Research and receives research funding from Deutsche Forschungsgemeinschaft and reports grants from Biogen outside the submitted work. J. Poettgen reports grants from Deutsche Rentenversicherung Bund outside the submitted work. SM Gold receives research funding from Deutsche Forschungsgemeinschaft, Bundesministerium für Bildung und Forschung, and the National MS Society. A. Fischer and L. Schaefer have nothing to declare.
