# Supplementary figures and images for: Impairment and restrictions in possibly benign multiple sclerosis
Source: Brain Behav. 2019 Mar 18;9(4):e01259. doi: 10.1002/brb3.1259 (PMC6456783; doi:10.1002/brb3.1259)

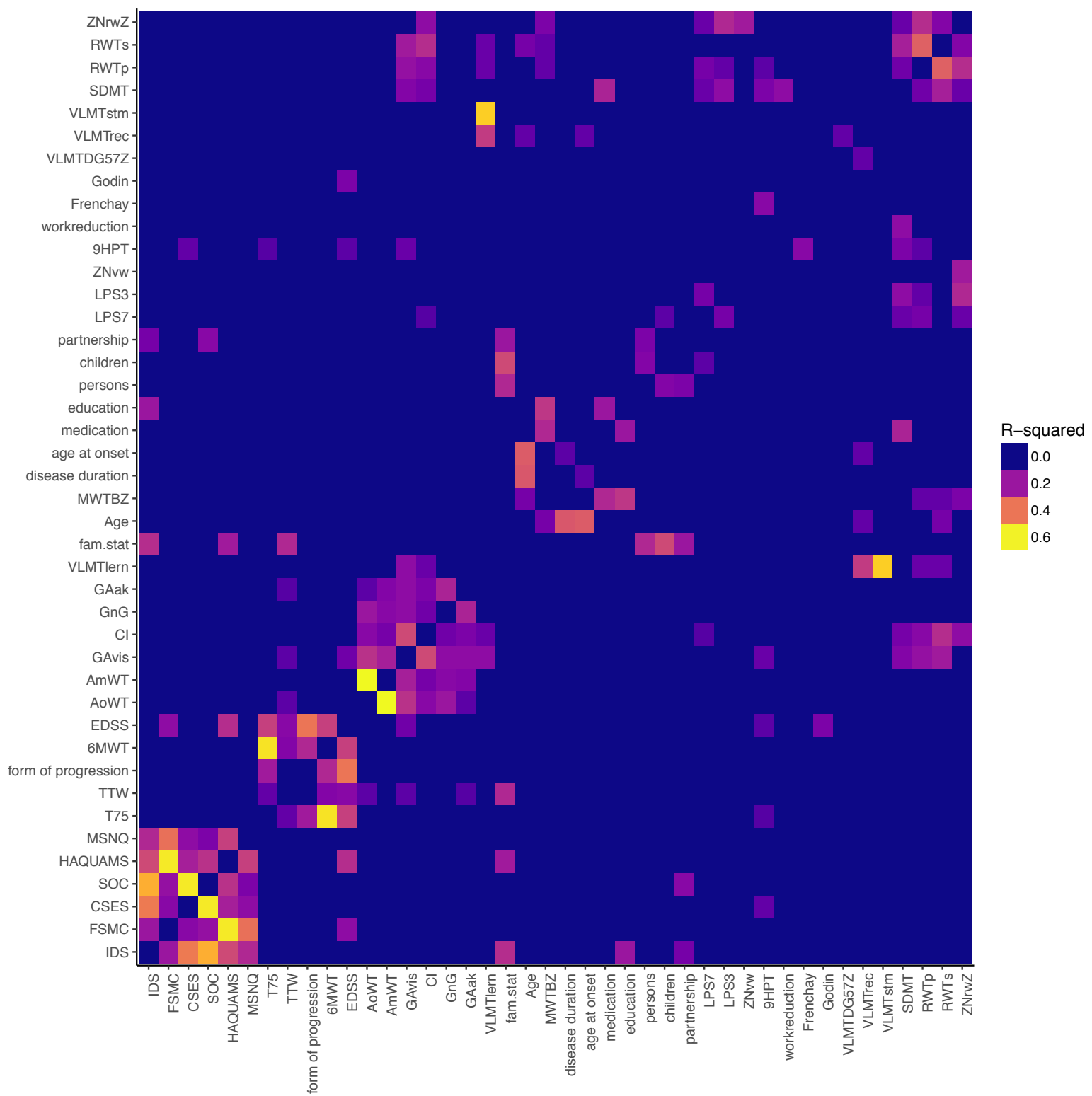

Supplement: Supplementary file 1 [file BRB3-9-e01259-s001.pdf]
