# Supplementary material for: Impairment and restrictions in possibly benign multiple sclerosis
Source: Brain Behav. 2019 Mar 18;9(4):e01259. doi: 10.1002/brb3.1259 (PMC6456783; doi:10.1002/brb3.1259)
Supplement: Supplementary file 2 [file BRB3-9-e01259-s002.docx]

**Supplementary**

**Table S1**: **References for neuropsychological tests**

| VLMT | Helmstaedter C, Lendt M, Lux S. Verbaler Lern- und Merkfähigkeitstest. *Diagostica* 1999; 45: 205–211 |
| --- | --- |
| ZN | Oswald WD, Fleischmann UM. *‘Das Nürnberger-Alters-Inventar (NAI) Hogrefe’*. 1997. |
| TAP | Zimmermann P, Fimm B, Fimm V, et al. Testbatterie zur Aufmerksamkeitsprüfung Version 2.3 Peter Zimmermann & Bruno Fimm. |
| SDMT | Smith A. The symbol digit modalities test. *Learn Dis* 1968; 3: 83–91. |
| RWT | Aschenbrenner S, Kucha O, Lange K. *Regensburger Wortflüssigkeitstest: RWT*. Göttingen: Hogrefe, Verlag für Psychologie, 2000. |
| LPS 3and 7 | Horn W. *Leistungsprüfsystem: LPS*. Verlag für Psychologie, Hogrefe, 1983. |

| **Table S2: Social status** | n | **(**%**)^1^** |  |
| --- | --- | --- | --- |
| **education** |  |  |  |
| >10y | 55 | (59%) |  |
| ≤10y | 38 | (41%) |  |
| **family status** |  |  |  |
| married | 60 | (65%) |  |
| single | 23 | (25%) |  |
| separated | 7 | (8%) |  |
| widowed | 2 | (2%) |  |
| partnership | 70 | (75%) |  |
| No partnership | 21 | (25%) |  |
| **children** |  |  |  |
| >=1 child | 55 | (59%) |  |
| Mean (SD) | 1.14 | (1.17) |  |
|  |  |  |  |
| **employment** |  |  |  |
| fulltime | 33 | (35.5%) |  |
| halftime | 19 | (20.4%) |  |
| pension | 8 | (8.6%) |  |
| disability pension | 19 | (20.4%) |  |
| housewife | 4 | (4.3%) |  |
| unemployed | 5 | (5.4%) |  |
| education | 1 | (1.1%) |  |
| other | 4 | (4.3%) |  |

^1^Frequencies and percent if not other indicated

| **Table S 3**  **Frenchay activity index** |  | | **Current cohort** | **Einarsson et al. 2006** |  |
| --- | --- | --- | --- | --- | --- |
| n= |  | | 94 | 164 |  |
| Question (Code in %) |  | |  |  |  |
| 1 Preparing Meals (0/1/2/3)^†^ |  | | 12/  7/ 22/  59 | 34/  6/ 11/ 49 |  |
| 2 Washing up after meals (0/1/2/3)^†^ |  | | 7/  8/  17/  69 | 29/  1/ 21/ 49 |  |
| 3 washing clothes (0/1/2/4)^‡^ |  | | 10/  6/ 11/  74 | 38/  2/  9/ 51 |  |
| 4 light housework (0/1/2/4)^‡^ |  | | 2/  7/ 14/ 77 | 32/  3/ 12/ 53 |  |
| 5 heavy housework (0/1/2/4)^‡^ |  | | 13/ 21/ 29/ 37 | 61/  4/  8/ 26 |  |
| 6 local shopping (0/1/2/4)^‡^ |  | | 4/  2/ 10/ 84 | 34/  2/ 10/ 54 |  |
| 7 social occasions(meeting friends, cinema…) (0/1/2/4)^‡^ |  | | 2/ 19/ 35/ 44 | 14/ 16/ 31/ 39 |  |
| (8) walk outside for more than 15 minutes (0/1/2/4)^‡^ | | | 1/  7/ 62/ 31 | 59/  2/   6/ 33 |  |
| 9 actively pursuing hobby (0/1/2/4)^‡^ | |  | 15/ 13/ 12/ 58 | 49/  2/  9/ 40 |  |
| 10 driving car/going on bus (0/1/2/4)^‡^ | |  | 2/  6/  6/  86 | 43/  2/  5/ 49 |  |
| 11 travel outing/ car ride (0/1/2/4)^§^ | |  | 21/ 41/ 25/ 13 | 34/ 32 /24/  9 |  |
| 12 gardening (0/1/2/3)^¶^ | |  | 40/ 18/ 15/ 28 | 68/ 12/ 12/  9 |  |
| 13 household maintenance (0/1/2/3)^¶^ | |  | 4/ 11/ 20/ 65 | 59/ 19/ 14/  8 |  |
| 14 reading books (0/1/2/3)^††^ | |  | 12/14/14/59 | 38/ 17/ 29/ 17 |  |
| 15 gainful work(0/1/2/3)^‡‡^ | |  | 33/  8/ 25/ 34 | 58/ 2/20/21 |  |
|  | | | | | |

^†^ in the last 3 months (0: never; 1: <1x/week; 2: 1-2 x/ week; 3: during the most days)

^‡^ in the last 3 months (0: never; 1:1-2 x in 3 months; 2: 3-12x in 3 months; 4:at least 1x/week)

^§^ in the last 6 months (0: never, 1: 1-2x in 6 months; 2: 3-12x in 6 months; 4: at least every 14 days)

^¶^in the last 6 months (0: never, 1: 1-2x in 6 months; 2: 3-12x in 6 months; 3: at least every 14 days)

^††^in the last 6 months (0: never, 1: 1x in 6 months; 2: <14 days in 14 days; 3 > 1x in 14 days)

^‡ ‡^ in the last 6 months (0: never, 1: up to 10 hours/ week; 2: 10-30 hours per weeks; 3: at least 30 hours per week)

**Supplemental figure: Association matrix of outcomes**

Color scale indicates the strength of the association assessed with R^2^. Zero values (blue) indicate non-significance after FDR correction. R2-values from significant Fisher’s exact tests were set to 0.2 for plotting.

fam stat= family status, persons= persons living at home, ZNfw/ZNrwZ= repeating numbers forward/backward, RWTs/ RWTp= semantic/ phonematic word fluency, VLMTstm= memory span, VLMTSlern= lerning, VLMTDG5-7Z= remembering, VLMTrec= recognition, GAak/ GAvis= divided attention acustic/visual, GnG= Selective attention, AmWT/AoWT= tonic/phasic alertness, MWTBZ (not included in the study).

cogMean = computed mean z-score of all neuropsychological tests. 9HPT = Nine-Hole Peg Test, CSES = Coping Self-Efficacy Scale, SOC = Sense Of Coherence, IDS= Quick Inventory of Depressive Symptomatology, QIDS-SR16, MSNQ = Multiple Sclerosis Neuropsychological Questionnaire, HAQUAMS= Hamburg Quality of Live Scale in Multiple Sclerosis, FSMC = Fatigue Scale for Motor and Cognitive Functions, EDSS = Expanded Disability Status Scale, TTW = Timed Tandem Walk, T75 = 25-Foot Walk, Godin = Godin leisure time activities.
